# Universal screening of Tanzanian HIV-infected adult inpatients with the serum cryptococcal antigen to improve diagnosis and reduce mortality: an operational study

**DOI:** 10.1186/1758-2652-14-48

**Published:** 2011-10-11

**Authors:** Bahati MK Wajanga, Samuel Kalluvya, Jennifer A Downs, Warren D Johnson, Daniel W Fitzgerald, Robert N Peck

**Affiliations:** 1 Department of Medicine, Bugando Medical Centre, Box 1370, Mwanza, Tanzania; 2 Department of Medicine, Weill Bugando University College of Health Sciences, Mwanza, Tanzania; 3 Center for Global Health, Department of Medicine, Weill Cornell Medical College, York Avenue, New York, New York, USA

## Abstract

**Background:**

Cryptococcal meningitis is a leading cause of death among HIV-infected individuals in sub-Saharan Africa. Recent developments include the availability of intravenous fluconazole, cryptococcal antigen assays and new data to support fluconazole pre-emptive treatment. In this study, we describe the impact of screening HIV-positive adult inpatients with serum cryptococcal antigen (CRAG) at a Tanzanian referral hospital.

**Methods:**

All adults admitted to the medical ward of Bugando Medical Centre are counseled and tested for HIV. In this prospective cohort study, we consecutively enrolled HIV-positive patients admitted between September 2009 and January 2010. All patients were interviewed, examined and screened with serum CRAG. Patients with positive serum CRAG or signs of meningitis underwent lumbar puncture. Patients were managed according to standard World Health Organization treatment guidelines. Discharge diagnoses and in-hospital mortality were recorded.

**Results:**

Of 333 HIV-infected adults enrolled in our study, 15 (4.4%) had confirmed cryptococcal meningitis and 10 of these 15 (66%) died. All patients with cryptococcal meningitis had at least two of four classic symptoms and signs of meningitis: fever, headache, neck stiffness and altered mental status. Cryptococcal meningitis accounted for a quarter of all in-hospital deaths.

**Conclusions:**

Despite screening of all HIV-positive adult inpatients with the serum CRAG at the time of admission and prompt treatment with high-dose intravenous fluconazole in those with confirmed cryptococcal meningitis, the in-hospital mortality rate remained unacceptably high. Improved strategies for earlier diagnosis and treatment of HIV, implementation of fluconazole pre-emptive treatment for high-risk patients and acquisition of better resources for treatment of cryptococcal meningitis are needed.

## Background

Cryptococcal meningitis is one of the most common and severe opportunistic infections among people infected with HIV: there are an estimated 720, 000 cases and 500, 000 deaths per year in sub-Saharan Africa alone [1,2]. In community-based studies, cryptococcal meningitis accounts for between 13% and 44% of all deaths of HIV-infected individuals [3-5]. Despite the roll out of antiretroviral therapy (ART), the incidence of cryptococcal meningitis remains at about 3% per year among HIV-infected individuals in sub-Saharan Africa [2,6].

Although advances in the treatment of cryptococcal meningitis have decreased mortality in high-income countries, mortality due to cryptococcal meningitis in middle- and low-income countries remains high [2,7,8]. The mortality rate from cryptococcal meningitis in sub-Saharan Africa has been estimated at 70% compared with 55% in other low- and middle-income countries and 20% in high-income countries [2]. This higher mortality is thought to be related to delayed diagnosis of both HIV and cryptococcal meningitis, as well as the inaccessibility of first-line treatment with combination amphotericin/flucytosine induction chemotherapy and intensive intracranial pressure management [8,9].

Our referral hospital in northwestern Tanzania has recently obtained two tools in an effort to improve management of cryptococcal meningitis: intravenous fluconazole and the cryptococcal antigen assay. High-dose fluconazole (800-1200 mg daily) has been recommended as an alternative, though suboptimal, induction therapy for regions where amphotericin and/or flucytosine are not available [8,10-12]. The serum cryptococcal antigen assay is a sensitive and specific screening tool that has been studied in several outpatient HIV-infected cohorts in sub-Saharan Africa, where the prevalence has been 7% to 8% [13,14]. The prevalence of cryptococcal antigenemia among HIV-positive inpatients in sub-Saharan Africa has not been reported.

In our hospital, we have been tracking the diagnosis and outcomes of cryptococcal meningitis since January 2010. In the nine months before the initiation of this study, between January and August 2010, there were a total of 47 cases of cryptococcal meningitis admitted to our hospital (5.2 cases per month) and 34 of 47 (72.3%) of these patients died in hospital despite treatment with high-dose intravenous fluconazole. Clinicians felt that delayed diagnosis, often one to two weeks after the day of admission, may have been contributing to this mortality and that earlier diagnosis and earlier initiation of IV fluconazole, closer to the time of admission, may improve the outcome of these patients.

Therefore, in this study we screened a population of HIV-positive adult inpatients with the serum cryptococcal antigen in order to determine if universal screening at the time of admission could lead to earlier diagnosis and treatment of cryptococcal meningitis and to better in-hospital outcomes. We also describe the prevalence, clinical characteristics and in-hospital mortality of cryptococcal meningitis treated with high-dose intravenous fluconazole in the ART era.

## Methods

### Trial design and study participants

This prospective cohort study was completed between September 2009 and January 2010 in the inpatient medical wards of Bugando Medical Centre (BMC). BMC is a tertiary referral hospital that serves the Lake Victoria region of northwestern Tanzania (population of about 13 million) and is located in the city of Mwanza. On average, 10 adult patients are admitted to our medical wards daily. Approximately 25% are HIV positive. By hospital policy, all patients who are not known to be HIV positive undergo counselling for HIV at the time of admission and are tested for HIV if they consent.

All HIV-positive adults admitted to the medical ward during the study period, who met the enrolment criteria and signed informed consent, were enrolled in the study. Patients younger than 18 years, those who had previously been diagnosed with cryptococcal meningitis and those who had received pre-emptive treatment for cryptococcal meningitis were excluded.

### Data collection

Patients were interviewed and examined within 24 hours of admission using a structured questionnaire to collect demographic information, clinical symptoms and physical signs. For all HIV-infected patients, 5 milliliters of blood was drawn for serum cryptococcal antigen at the time of enrolment, as well as for CD4 cell count if this had not been documented within the last three months. Lumbar puncture was performed on all patients with signs of clinical meningitis or positive serum cryptococcal antigen. Cerebrospinal fluid (CSF) was sent for both cryptococcal antigen and India ink staining. The cryptococcal antigen assay is a World Health Organization (WHO) approved test that was already being used routinely for diagnosis of cryptococcal meningitis at BMC before the beginning of this study. The results of all tests were reported immediately to the responsible clinicians. Discharge diagnoses and outcome were recorded for all patients.

The treatment of cryptococcal meningitis followed the recommendations of WHO and the Tanzanian Ministry of Health [15-17]. Pre-emptive treatment for cryptococcal meningitis is not yet recommended in Tanzania and none of our patients had received pre-emptive treatment. All patients with cryptococcal meningitis in this study were treated with two weeks of intravenous fluconazole (1200 mg daily, intensive phase) followed by eight weeks of oral fluconazole (400 mg daily, maintenance phase). Patients whose physicians judged their CSF drip rate to be increased at the time of the initial lumbar puncture received serial lumbar punctures for reduction of intracranial pressure. Manometers to quantify CSF pressure were not available in our hospital.

### Laboratory analyses

Serum and CSF cryptococcal antigen assay was performed using the latex agglutination test kit (CALAS, Meridian Bioscience Europe, Nice, France) following manufacturer instructions. This assay includes a pronase and has been shown to have a sensitivity of 93% to 100% and a specificity of 96% to 98% [18]. Serial dilutions were performed to determine quantitative titers. CSF cryptococcal antigen assays with a titre of ≥1:4 were defined as positive. Due to limited supply of reagent, serum CRAG titres were only diluted to 1:64 and CSF CRAG titres were only diluted to 1:32. CSF was also examined with India ink stain (Pelikan, Hanover, Germany). CD4 counts were determined using FACSCalibur Flow Cytometry (BD, San Jose, USA). Fungal culture was not performed.

### Definitions

Cryptococcal meningitis was defined as a positive cryptococcal antigen and/or India ink test in the CSF. Disseminated cutaneous cryptococcosis was defined as a positive cryptococcal antigen with consistent skin lesions since our hospital was not equipped for performance of confirmatory skin biopsies. The diagnoses of tuberculosis, non-cryptococcal meningitis, chronic diarrhea, pneumocystis pneumonia and other opportunistic conditions were made according to WHO clinical definitions [15,16].

### Data analysis

Data were entered into Microsoft Excel 2007 and were analyzed using SAS (Cary, North Carolina). Categorical variables were summarized by frequency and percentage, and continuous variables were summarized by median and interquartile range. Categorical variables were compared using the Chi-squared (χ2) or Fisher's Exact tests and continuous variables were compared using the Log Rank-Sum test. All variables with significant associations to cryptococcal meningitis on univariate analysis were subjected to multivariate analysis. All statistical testing was done at the 95% confidence interval, and we considered a p value < 0.05 to be statistically significant.

### Ethical issues

Ethical approval was obtained from the BMC and Weill Cornell Medical College IRBs and a written informed consent was obtained from each patient or their surrogate for unconscious patients. Due to limited ability to communicate with family members who did not visit the hospital, surrogates were chosen from among the people who cared for the unconscious patient in the hospital. Primary relatives and/or spouses were preferred and used in almost all cases. HIV status was not revealed to surrogates who were not previously aware of the HIV status of the patient.

## Results

### Enrolment

**Figure 1:**
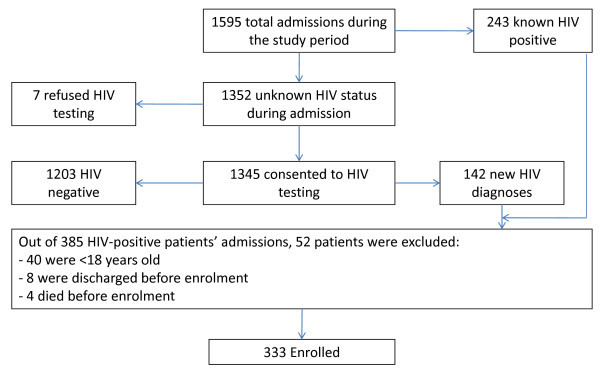
**Screening and enrollment**. Screening and enrolment statistics for 1595 adults admitted to the medical wards of Bugando Medical Centre in Mwanza, Tanzania between 1 September 2009 and 9 January 2010

Between 1 September 2009 and 9 January 2010, a total of 1595 adults were admitted to the BMC medical wards. Of these, 243 (15.2%) were known to be HIV positive at the time of admission and 142 (8.9%) were found to be HIV positive after voluntary counseling and testing. Thus a total of 385 HIV-positive adults were admitted to BMC during the study period, and 333 (86.5%) were enrolled. Screening, exclusion and enrolment statistics are summarized in Figure 1.

### Patient characteristics

Among the 333 adult HIV-positive patients enrolled in the study, the median age was 36 years (IQR 18-54 years) and 53.8% of the patients were women. As noted, 243 (73.0%) were aware of their HIV status on admission. The median CD4 count was 209 cells/mm^3 ^(IQR 87-378) and 93 patients (27.9%) had CD4 counts of less than 100 cells/mm^3^. Among the patients enrolled, 164 (49.3%) were already on ART, and the majority of these (70.7%) had been on ART for 180 days or more.

### Screening for cryptococcal meningitis

Of the 333 HIV-infected adult inpatients enrolled in this study, 17 (5.1%) had a positive serum cryptococcal antigen. Among these 17 patients, the median CD4 count was 68 cells/mm^3 ^(IQR 41-87, range 1-102 cells/mm^3^). Fifteen of these 17 patients (4.4% of all study patients) had confirmed cryptococcal meningitis with both a positive CSF cryptococcal antigen and a positive India ink test. The remaining two patients with positive serum but negative CSF cryptococcal antigen and India ink had skin lesions that were consistent with disseminated cutaneous cryptococcosis. No patient with a positive CSF cryptococcal antigen or India ink had a negative serum cryptococcal antigen.

Among the 15 patients with confirmed cryptococcal meningitis, the median age was 41 years (IQR 32-47) and 53.3% were women. Of these, nine (60%) knew their HIV status at the time of admission and six (40%) were on ART. The median CD4 count of patients with cryptococcal meningitis was 68 cells/mm^3 ^(IQR 54-87) and 14 (93.3%) had CD4 counts below 100 cells/mm^3 ^(overall range 1-102 cells/mm^3^). The prevalence of cryptococcal meningitis was 14 of 93 (15.0%) patients with CD4 counts below 100 cells/mm^3^, six of 90 (6.7%) with a new diagnosis of HIV, and five of 48 (10.4%) who had been on ART for less than three months. The baseline characteristics of our cohort are described in Table 1, divided by diagnosis of cryptococcal meningitis or other diagnosis.

**Table 1:** Baseline characteristics of HIV-infected adults admitted to Bugando Medical Centre divided by diagnosis

	Cryptococcal meningitis (n = 15)	Other diagnoses (n = 318)	p value
Age (years)			
18-30	3 (20.0%)	85 (26.7%)	0.77

31-40	4 (26.7%)	118 (37.1%)	0.59

41-50	5 (33.3%)	70 (22.01%)	0.34

51-60	2 (13.3%)	30 (9.4%)	0.65

> 60	1 (6.7%)	15 (4.7%)	0.53

Median (IQR)	41.0 (32-47)	36.0 (30-44)	0.29

**Gender**			

Male	7 (46.7%)	147 (46.2%)	1

Female	8 (53.3%)	171 (53.8%)	

**CD4 profile (cells/mm**^**3**^**)**			

< 100	14 (93.3%)	79 (24.8%)	< 0.0001

100-200	1 (6.7%)	61 (19.2%)	0.32

> 200	0 (0%)	178 (56.0%)	< 0.0001

Median (IQR)	68.0 (54-87)	228.0 (101-379)	0.0006

**HIV status on admission**			

New diagnosis	6(40%)	84(26.42%)	0.25

Known	9(60%)	234(73.58%)	

**Days on antiretroviral therapy**			

Never	8 (53.3%)	162 (50.9%)	1

< 180	5 (33.3%)	43 (13.5%)	0.05

> 180	2 (13.3%)	113 (35.5%)	0.097

Median (IQR)	92 (61-214)	293 (147-589)	0.003

**Symptoms/signs on admission**			

Headache	11 (73.3%)	87 (27.6%)	0.0004

Fever (T > 37.5°C)	14 (93.3%)	204 (64.2%)	0.02

Altered mental status (GCS ≤14)	10 (66.7%)	19 (6.0%)	< 0.0001

Neck stiffness	9 (60%)	22 (6.9%)	0.0001

None of the above	0	97 (30.5%)	

≥ 1 of the above	15 (100%)	221 (69.5%)	0.007

≥ 2 of the above	15 (100%)	108 (33.9%)	< 0.0001

### Clinical characteristics of cryptococcal meningitis

The univariate analysis for baseline clinical characteristics that predicted the diagnosis of cryptococcal meningitis is shown in Table 1. Significant predictors by univariate analysis included CD4 counts of less than 100 cells/mm^3^, less than 180 days on ART, headache, fever (> 37.5°C), altered mental status (Glascow Coma Scale ≤ 14) and neck stiffness. By multivariate analysis, risk factors for cryptococcal meningitis included: CD4 counts of less than 100 cells/mm^3 ^(OR 28.0, 95% CI 2.9-272.0), altered mental status (OR 25.3, 95% CI 5.1-126.2), neck stiffness (OR 10.2, 95% CI. 2.2-46.5) and fever (OR 5.6, 95% CI 1.1- 29.1). All patients had increased intracranial pressure as measured by the drip rate at the time of initial, diagnostic lumbar puncture, and all patients underwent serial drainage of 10-15 mL of CSF on hospital days 0, 3 and 7 according to our hospital's protocol. All patients with cryptococcal meningitis had at least two of the four classic symptoms and signs of meningitis (fever, headache, altered mental status and neck stiffness).

### Outcomes

Of the 333 HIV-infected adult inpatients in our study, 38 (11.4%) died in hospital. Of the 15 with cryptococcal meningitis, 10 died. Cryptococcal meningitis accounted for 26.3% (10 of 38) of all deaths, more than any other single diagnosis. The discharge diagnoses, in-hospital mortality rates and contribution to overall mortality are summarized in Table 2.

**Table 2:** Discharge diagnoses and in-hospital mortality of HIV-infected adults admitted to Bugando Medical Centre

Diagnosis	Frequency	Mortality rate	% of overall mortality
Malaria	53/333 (15.9%)	1/53 (1.9%)	1/38 (2.6%)

Tuberculosis	36/333 (10.8%)	1/36 (2.8%)	1/38 (2.6%)

Non-cryptococcal meningitis	31/333 (9.3%)	9/31 (29%)	9/38 (23.7%)

Chronic diarrhea	25/333 (7.5%)	1/25 (4%)	1/38(2.6%)

Bacterial pneumonia	21/333 (6.3%)	1/21 (4.8%)	1/38 (2.6%)

Anemia	11/333 (3.3%)	1/11 (9.1%)	1/38 (2.6%)

**Cryptococcal meningitis**	**15/333 (4.5%)**	**10/15 (66.7%)**	**10/38 (26.3%)**

Pneumocystis pneumonia	14/333 (4.2%)	3/14 (21.4%)	3/38 (7.9%)

Schistosomiasis	13/333 (3.9%)	2/13 (15.4%)	2/38 (5.3%)

Acute diarrhea	11/333 (3.3%)	0	0

**Other diagnoses (deaths listed in parentheses): **Esophageal candidiasis 9 (0), Urinary tract infections 9 (0), Kaposi's sarcoma 8 (0), Peripheral neuropathy 8 (0), Peptic ulcer diseases 5 (0), Spontaneous bacteria peritonitis 7 (0), Deep vein thrombosis 5 (0), Abscesses 5 (1), Pleural effusion 5 (0), Hypertension 4 (0), Wasting syndrome 3 (1), HIV-associated nephropathy 3 (0), Encephalopathy 3(0), Diabetic mellitus 2 (0), Skin cryptococcal infection 2 (0), Peripartum cardiomyopathy 2 (0), Stroke 2 (1), Viral hepatitis 2 (0), Herpes zoster 1 (0), Hodgkin's lymphoma 1 (1), Aspiration pneumonia 1 (0), Dysentery 4 (1), Post-abortal sepsis 1 (1), Cellulitis 1 (0), Cerebral toxoplasmosis 1 (0), Osteomyelitis 1 (0), Rheumatoid arthritis 1 (0), Bilateral renal tumor 1 (1), Stevens Johnson syndrome 1 (0), Genital warts 1 (0), Spontaneous pneumothorax 1 (1), Transverse myelitis 1 (0), Empyema thoracic 1 (1), Pericardial effusion 1 (0)

Mortality was associated with higher serum and CSF cryptococcal antigen titers. Of the 11 patients with CSF antigen titre ≥ 1:32 or serum titre ≥ 1:64 mortality was 10/11, as compared with 0/4 for those with lower titres (p = 0.004). Serum and CSF cryptococcal antigen titres were the only statistically significant predictors of death among patients with cryptococcal meningitis in our cohort.

## Discussion

Of the 333 HIV-infected adults consecutively admitted to our Tanzanian hospital during the ART era and screened with the serum cryptococcal antigen, 15 (4.5%) had confirmed cryptococcal meningitis and 10 of 15 died in hospital despite high-dose intravenous fluconazole initiated within 24 hours of admission. All patients with cryptococcal meningitis had typical symptoms. One-quarter of all deaths in this cohort were due to cryptococcal meningitis.

The prevalence of cryptococcal meningitis among HIV-infected adult inpatients may be decreasing with earlier HIV diagnosis and increasing ART use. We observed a lower prevalence of cryptococcal meningitis (4.5%) than prior studies. Another study conducted at a referral hospital (Kilimanjaro Christian Medical Center) in central Tanzania reported a prevalence of cryptococcal meningitis of 40 out of 149 (26.8%) HIV-infected adults admitted with a chief complaint of either headache or altered mental status [19]. Of 113 patients with either headache or altered mental status in our cohort, 15 (13.3%) had cryptococcal meningitis. The lower prevalence of cryptococcal meningitis in our population is likely due to higher median CD4 count (209 vs. 147 cells/mm^3 ^at KCMC) as well as the inclusion of more patients on ART (50% vs. 22% at KCMC) [20].

Despite the low prevalence of cryptococcal meningitis among the HIV-positive adult inpatients in our study and the high rates of ART use (50% in our cohort), cryptococcal meningitis still accounted for 26% of all in-hospital AIDS deaths. These findings are consistent with reports from community-based, HIV-infected adult cohorts in sub-Saharan Africa during both the pre-ART and post-ART eras where 13% to 44% of deaths were attributed to this infection [2-6]. The in-hospital mortality rates for cryptococcal meningitis also remain high despite recent improvements in access to high-dose intravenous fluconazole and cryptococcal antigen testing [8,9,21]. Although patients were diagnosed and initiated on fluconazole within 24 hours of presentation to our hospital, the mortality rate remained close to 70%, comparable with rates reported by others in sub-Saharan Africa [2,10,11].

The 66% in-hospital mortality rate seen in our patients with cryptococcal meningitis is very high compared with 20% acute mortality rates seen in high-income countries where amphotericin-based induction therapy, intensive intracranial pressure management and earlier presentation are the norm [2]. Intravenous fluconazole is known to be inferior to combination induction therapy with amphotericin/flucytosine [9,11]. Also, our hospital does not have the equipment necessary for intensive intracranial pressure management, which has been associated with decreased mortality in cryptococcal meningitis and is recommended by the Infectious Diseases Society of America [12,22]. One possible benefit of the early, targeted screening of inpatients with the serum CRAG is that early, aggressive, empiric ICP management could be initiated on CRAG-positive patients according to the protocol recommended by Bicanic *et al *[23]. Finally, patients in our setting often present for medical care late in the course of their illness [24,25].

Universal screening of symptomatic, HIV-infected adult inpatients with a serum cryptococcal antigen does not seem to be necessary. All of the patients with cryptococcal meningitis in our cohort had at least two of the four classic symptoms and signs of meningitis: headache, fever, neck stiffness and altered mental status. Predictors of cryptococcal meningitis by multivariate analysis included CD4 T cell counts of under 100 cells/mm^3^, altered mental status (GCS ≤ 14), fever (temperature > 37.5C) and neck stiffness, consistent with other studies [3,19,26]. Among adult HIV-infected inpatients, targeted serum CRAG screening for patients with symptoms and signs of meningitis and (if known) a CD4 T cell count of less than 200 cells/mm^3 ^may be a reasonable approach in hospitals in sub-Saharan Africa.

A growing body of research suggests that an even better use of the serum CRAG would be as a screening tool among asymptomatic HIV-infected adults, particularly before the initiation of ART in patients with CD4 counts under 100 cells/mm^3 ^[13,27]. In patients found to have asymptomatic antigenemia, pre-emptive treatment with fluconazole has been shown to reduce mortality [14,28]. One of the limitations of our study is that we could not detect patients with asymptomatic antigenemia since we only screened symptomatic, hospitalized patients.

Of the 15 cases of cryptococcal meningitis in our study, five (33%) occurred in adults who had been on ART for less than three months and qualify as "ART-associated cryptococcosis" according to new consensus definitions [29]. We suspect that some if not all of these cases represent unmasking cryptococcal meningitis Immune Reconstitution Inflammatory Syndrome (IRIS), but cannot definitively make this diagnosis due to absence of baseline cryptococcal investigations in our patients. This is a limitation of our study.

Based on these study results, our hospital has been able to preserve resources by targeting CRAG testing among inpatients to those with concerning symptoms and CD4 counts under 200 cells/mm^3^. Our hospital has also prioritized the pursuit of other measures to reduce mortality due to cryptococcal meningitis, including improved strategies for earlier diagnosis and treatment of HIV, implementation of fluconazole pre-emptive treatment protocols for high-risk patients, and acquisition of better resources for treatment of cryptococcal meningitis.

## Conclusions

This study shows how, despite the roll out of ART and the increased availability of fluconazole and cryptococcal antigen assays, cryptococcal meningitis still accounted for a quarter of the deaths of HIV-infected adult inpatients in sub-Saharan Africa. Patients with cryptococcal meningitis presented with typical symptoms, and universal screening with a serum cryptococcal antigen assay at the time of admission did not seem to improve diagnosis rates compared with traditional, symptom and CD4 count guided testing. Despite immediate treatment with high-dose intravenous fluconazole, two out of three patients died in hospital. These findings point to the urgent need for better strategies and tools for the prevention and treatment of cryptococcal meningitis in sub-Saharan Africa.

## Competing interests

The authors declare that they have no competing interests.

## Authors' contributions

BW and RP participated in study design, coordination, data collection, data analysis and drafting of the manuscript. SK participated in study design, coordination and drafting of the manuscript. JD and DF contributed to data analysis and drafting of the manuscript. WJ drafted the manuscript. All authors read and approved the final manuscript.
